# Prebiotic dietary fibre intervention improves fecal markers related to inflammation in obese patients: results from the Food4Gut randomized placebo-controlled trial

**DOI:** 10.1007/s00394-021-02484-5

**Published:** 2021-02-05

**Authors:** Audrey M. Neyrinck, Julie Rodriguez, Zhengxiao Zhang, Benjamin Seethaler, Cándido Robles Sánchez, Martin Roumain, Sophie Hiel, Laure B. Bindels, Patrice D. Cani, Nicolas Paquot, Miriam Cnop, Julie-Anne Nazare, Martine Laville, Giulio G. Muccioli, Stephan C. Bischoff, Jens Walter, Jean-Paul Thissen, Nathalie M. Delzenne

**Affiliations:** 1 Metabolism and Nutrition Research Group, Louvain Drug Research Institute, UCLouvain, Université catholique de Louvain, avenue E. Mounier box B1.73.11, B-1200 Brussels, Belgium; 2 Department of Medicine, University of Alberta, Edmonton, Canada; 3 Institute of Nutritional Medicine, University of Hohenheim, Hohenheim, Germany; 4 Bioanalysis and Pharmacology of Bioactive Lipids Research Group, Louvain Drug Research Institute, UCLouvain, Université catholique de Louvain, Brussels, Belgium; 5 WELBIO- Walloon Excellence in Life Sciences and Biotechnology, UCLouvain, Université catholique de Louvain, Brussels, Belgium; 6 Laboratory of Diabetology, Nutrition and Metabolic Disease, Université de Liège, Liège, Belgium; 7 ULB Center for Diabetes Research, Université Libre de Bruxelles, Brussels, Belgium; 8 Division of Endocrinology, Erasmus Hospital, Université Libre de Bruxelles, Brussels, Belgium; 9 Rhône-Alpes Research Center for Human Nutrition, Université-Lyon, CarMeN Laboratory, Hospices Civils de Lyon, Lyon, France; 10 Department of Medicine, and School of Microbiology, APC Microbiome Ireland, University College Cork, Cork, Ireland; 11 Pole of Endocrinology, Diabetes and Nutrition, Institut de Recherche Expérimentale et Clinique, UCLouvain, Université catholique de Louvain, Brussels, Belgium

**Keywords:** Gut microbiota, Obesity, Prebiotic, Microbial metabolites

## Abstract

**Purpose:**

Inulin-type fructans (ITF) are prebiotic dietary fibre (DF) that may confer beneficial health effects, by interacting with the gut microbiota. We have tested the hypothesis that a dietary intervention promoting inulin intake *versus* placebo influences fecal microbial-derived metabolites and markers related to gut integrity and inflammation in obese patients.

**Methods:**

Microbiota (16S rRNA sequencing), long- and short-chain fatty acids (LCFA, SCFA), bile acids, zonulin, and calprotectin were analyzed in fecal samples obtained from obese patients included in a randomized, placebo-controlled trial. Participants received either 16 g/d native inulin (prebiotic *n* = 12) *versus* maltodextrin (placebo *n* = 12), coupled to dietary advice to consume inulin-rich *versus* inulin-poor vegetables for 3 months, in addition to dietary caloric restriction.

**Results:**

Both placebo and prebiotic interventions lowered energy and protein intake. A substantial increase in *Bifidobacterium* was detected after ITF treatment (*q* = 0.049) supporting our recent data obtained in a larger cohort. Interestingly, fecal calprotectin, a marker of gut inflammation, was reduced upon ITF treatment. Both prebiotic and placebo interventions increased the ratio of tauro-conjugated/free bile acids in feces. Prebiotic treatment did not significantly modify fecal SCFA content but it increased fecal rumenic acid, a conjugated linoleic acid (*cis*-9, *trans*-11 CLA) with immunomodulatory properties, that correlated notably to the expansion of *Bifidobacterium* (*p* = 0.031; *r* = 0.052).

**Conclusions:**

Our study demonstrates that ITF-prebiotic intake during 3 months decreases a fecal marker of intestinal inflammation in obese patients. Our data point to a potential contribution of microbial lipid-derived metabolites in gastro-intestinal dysfunction related to obesity.

**ClinicalTrials.gov Identifier:**

NCT03852069 (February 22, 2019 retrospectively, registered).

**Supplementary Information:**

The online version contains supplementary material available at 10.1007/s00394-021-02484-5.

## Introduction

Recent studies have highlighted the role of gut dysbiosis in the etiology and pathogenesis of obesity-related metabolic disorders [1]. Despite the controversial role of gut dysbiosis as a cause of obesity in humans, numerous animal (and also human) studies suggest beneficial metabolic effects of gut-derived microbial metabolites that could be used in the prevention and treatment of obesity and related metabolic disorders. The analysis of short-chain fatty acids (SCFA) allowed to establish for the first time a molecular link between bacterial activity towards nutrients, and host physiology [2, 3]. Notably, acetate, propionate, and butyrate, produced upon microbial fermentation of carbohydrates and fibre, may influence the production of gut hormones (like glucagon-like peptide 1) by enteroendocrine L cells, thereby having a beneficial impact on metabolic functions, intestinal epithelial integrity, appetite and glucose homeostasis [4–7]. Other bacterial co-metabolites (i.e. metabolites produced from sequential microbial and host enzymes activities), like bile acids (BA), may also modulate gut endocrine function, metabolism, energy homeostasis and inflammation [8]. Obesity is associated with gut dysbiosis and changes of BA pool concentration and composition [8, 9]. The gut microbiota also produces long chain fatty acid (LCFA)-derived metabolites from dietary PUFA [10]. Some of these have conjugated double bounds (e.g. isomers of linoleic and linolenic acids) and can activate nuclear receptors playing key roles in the regulation of metabolism and inflammation. Those bacterial metabolites were also identified as potential anti-obesogenic agents [11–13].

The manipulation of the gut microbiome, which is largely influenced by the diet, appears as an innovative therapeutic tool to prevent or control obesity and related diseases. Of particular interest, some non-digestible dietary fibre (DF) called prebiotics, are fermented by the gut microbiota, thereby conferring potentially beneficial health effects [14, 15]. On the basis of numerous studies in animals and humans, it has been proposed that fermentable prebiotic DF might increase satiety, improve obesity-related metabolic disorders, and modulate gut-related immunity [16–20]. The mechanisms proposed to explain such effects often involve the bacterial metabolites such as SCFA. Inulin-type fructans (ITF) are prebiotic DF that promote *Bifidobacterium* spp. and produce SCFA upon fermentation; their administration may improve health outcomes, especially in the context of obesity [14, 21]. We previously conducted the multicenter FOOD4GUT intervention trial in obese patients with co-morbidities to better understand how ITF present in food could play a role on gut microbiota homeostasis and health [22]. More recently, in the context of the FiberTAG project, we set out to establish a set of biomarkers of bacterial co-metabolites and gut barrier function and link DF intake and gut-microbiota related health effects [23]. For this purpose, we explored a FOOD4GUT subcohort to study the link between prebiotic intake, gut microbial signature in terms of bacterial composition, the profile of key gut-derived metabolites, and fecal biomarkers related to gut barrier function and gut inflammation such as fecal zonulin and fecal calprotectin, respectively [24, 25]. Of particular interest, fecal zonulin was validated as a marker for gut permeability in the course of the FiberTAG project (unpublished data) whereas fecal calprotectin is widely used to assess gut inflammation [26].

## Materials and methods

### Intervention

The FOOD4GUT study was a 3-month-long, multicentric, single-blind, placebo-controlled trial. Recruitment, enrollment, randomization, sample size determination, inclusion and exclusion criteria, and outcomes have been previously described [22]. Participants were included for a period of 3 months and randomized to consume either 16 g/d native inulin (extracted from chicory root, Cosucra, Belgium) or 16 g/d maltodextrin (Cargill, Belgium). During the first week, patients were asked to ingest half the dose to allow adaptation to the fibre. Patients in the prebiotic and placebo arms were asked to prepare and consume recipes with vegetables rich in ITF or poor in ITF, respectively, in addition to dietary caloric restriction [22]. The participants met a dietitian before and monthly during the intervention. At baseline, the dietitian calculated energy expenditure of the participants in order to prescribe a hypocaloric diet corresponding to −30% of the calculated energy expenditure. At all visits, one-week recall questionnaires were completed to evaluate dietary intake. This 1-week recall questionnaire has been designed to include vegetables particularly rich in fructans, as previously reported [27]. As compared to 24 h-recall method or food diaries, this questionnaire is more rapid and easy to complete and meets the objective to focus on fructan and DF intake [28]. Participants received a cookbook with recipes based on vegetables either rich or poor in fructans and were advised to consume at least one meal proposed in the recipe per day. According to a previous study [27], we selected a list of vegetables enriched in fructans, including artichoke, asparagus, black radish, Brussels sprouts, butternut, cauliflower, celeriac, celery, endive, garlic, Jerusalem artichoke, leek, onion, parsnip, pumpkin, salsify, shallot, spaghetti squash, tuberous parsley, turnip and zucchini; artichokes, Jerusalem artichokes, onions and salsify being the most enriched vegetables. Patients in the placebo arm were asked to consume daily recipes based on vegetables poorly enriched in fructans including beans, cress, cucumber, eggplant, lettuce, lamb’s lettuce, mushroom, peas, pepper, spinach, Swiss chard and tomato. Participants prepared their own meals. All participants and research staff (excepted dietitians who provided dietary advices and recipes books) were blinded to the treatments. Fresh stool samples were available for 24 patients from the St Luc subcohort at baseline and after 3 months of dietary intervention and were stored immediately at −80 °C until analyses of SCFA, LCFA, BA, zonulin, calprotectin and gut microbiota. The measurement of fecal microbial-derived metabolites, markers related to gut integrity and inflammation were not initially scheduled in the trial design (NCT03852069-FOOD4GUT study). We evaluated those parameters a posteriori in the context of the FiberTAG project [23], after approval by the “Comité d’éthique Hospitalo-facultaire de Saint-Luc” and in accordance to the written informed consent provided by participants (leaving the possibility to use biological material for future research outside the context of the FOOD4GUT study).This study has been carried out in accordance with followed the ethical guidelines set out in the Declaration of Helsinki. All participants provided written informed consent in compliance with the European law 2001/20/CE guidelines, before inclusion. All authors had access to the study data and reviewed and approved the final manuscript. The trial protocol was published on protocols.io (dx.doi.org/10.17504/protocols.io.baidica6) and the trial was registered at ClinicalTrials.gov under identification number NCT03852069.

### Energy and nutrient intakes

Energy and nutrient intakes were calculated from the 1-week recall using the Nubel Pro program (Nubel asbl, Brussels, Belgium). Fructan intake as well as fructan content of the recipes were calculated using the FiberTAG repertoire detailing prebiotic (oligo) saccharides (including fructans) in food products [28].

### Fecal microbiome sequencing and analysis

Bacterial DNA was extracted from fecal samples using the QIAamp DNA Stool Mini Kit (QIAGEN, Hilden, Germany), as previously described [29]. The V5-V6 regions of the 16S rRNA gene of all samples were sequenced in the same run via by the MiSeq platform. The sequences and alpha and beta diversity indexes were calculated using QIIME2 [30]. An even depth of 10,769 sequences per sample was used to conduct microbiome diversity. Principal coordinates analysis (PCoA) plots of beta-diversity indexes were visualized using R software (ade4 package). Raw sequences are deposited into the Sequence Read Archive (SRA) of NCBI (http://www.ncbi.nlm.nih.gov/sra) and can be assessed with the accession number PRJNA669275.

### Markers of gut barrier and gut inflammation

Zonulin and calprotectin were measured in fecal samples using enzyme-linked immunosorbent assays (K5600; K6927; Immundiagnostik AG, Bensheim, Germany) as previously described [29].

### SCFA and LCFA analysis

SCFA and LCFA were analysed in fecal samples using gas chromatography with flame ionization detector as previously described [29].

### Bile acids analysis

BA were analyzed in fecal samples using a LTQ-Orbitrap mass spectrometer (ThermoFisher Scientific) coupled to an Accela HPLC system (ThermoFisher Scientific) as previously described [29].

### Statistical analysis

Data are expressed as mean ± SEM. Baseline data and between-group differences were analyzed by Mann–Whitney test. Within-group analyses were evaluated using a Wilcoxon paired test (from baseline to 3 months of intervention). Mixed model ANOVA followed by Sidak’s multiple comparisons test were performed for gastrointestinal symptoms to compare effects over time. For gut microbiota analysis, relative abundances performed in Qiime2 are expressed as percentage of mean of relative abundance and SEM, and were calculated on R for each taxon. At the genus level, if there were multiple taxa groups that all had the same genus name and belonged to the same family, we combined them together. The* p* value of the Wilcoxon test was adjusted for multiple testing with a 5% false discovery rate according to the Benjamini–Hochberg procedure (*q* value, significant if *q* < 0.05) [31]. Beta-diversity indices were evaluated on Qiime2 and visualized with a PcoA performed on R software, using ade4 package. A Monte Carlo rank test was assessed for beta-diversity based PcoA. Associations between the changes of bacteria significantly (Wilcoxon matched-pairs signed-rank tests) regulated by prebiotic intervention and the changes of gut-derived metabolites between month 3 and baseline were assessed by Spearman’s correlation tests. A significance level of *p* < 0.05 was adopted for all analyses. Heatmaps of correlation were visualized with the corrplot package on R software. Power estimations were calculated for the main outcome of the study (*Bifidobacterium* genus) and for the exploratory fecal parameters (including calprotectin) using the JMP Pro 14 software and taking into account the difference (change after 3 months from baseline value), standard deviation and sample size.

## Results

### Subject characteristics

One hundred and fifty subjects were randomized in the entire FOOD4GUT cohort [22]. Twenty-four patients from the St Luc hospital subcohort provided fresh fecal samples immediately frozen and stored at -80 °C (placebo *n* = 12, prebiotic *n* = 12). At baseline, the groups were similar in terms of clinical outcomes (Table S1). Anthropometric and cardiometabolic risk parameters were not significantly affected by the intervention in this subcohort.

### Nutrient intake

Both prebiotic and placebo interventions reduced energy and protein intake (Fig. S1, Table S2). Although carbohydrate intake was globally not affected by the intervention, we observed a lower sugar intake for the placebo group and a lower starch intake for the prebiotic group (between-variation *p* < 0.05). The lower fat intake (resulting from lower intake of SFA, n-3 PUFA, n-6 PUFA and trans-FA) was significant only in the placebo group. Of note, the baseline values of MUFA and PUFA (in particular n-6 PUFA) were not the same between both groups. Cholesterol intake was lower only with ITF treatment. The DF intake assessed by questionnaires (that does not consider the native inulin supplement) was not significantly modified in this subcohort. We calculated the average fructan content of recipes from both cookbooks using the FiberTAG repertoire detailing fructan content in food products [28]: it reached 11.2 ± 1.7 g per portion for the cookbook designed for prebiotic group *versus* 0.4 ± 0.1 g per portion for the cookbook designed for placebo group. Importantly, fructan intake estimated by using 1-week recall questionnaire was 3 times larger in the prebiotic group than the placebo group (independently of inulin supplement). Altogether, those results confirmed that the patients followed the dietetic advices throughout the intervention.

### Markers of gut barrier and gut inflammation

**Fig. 1:**
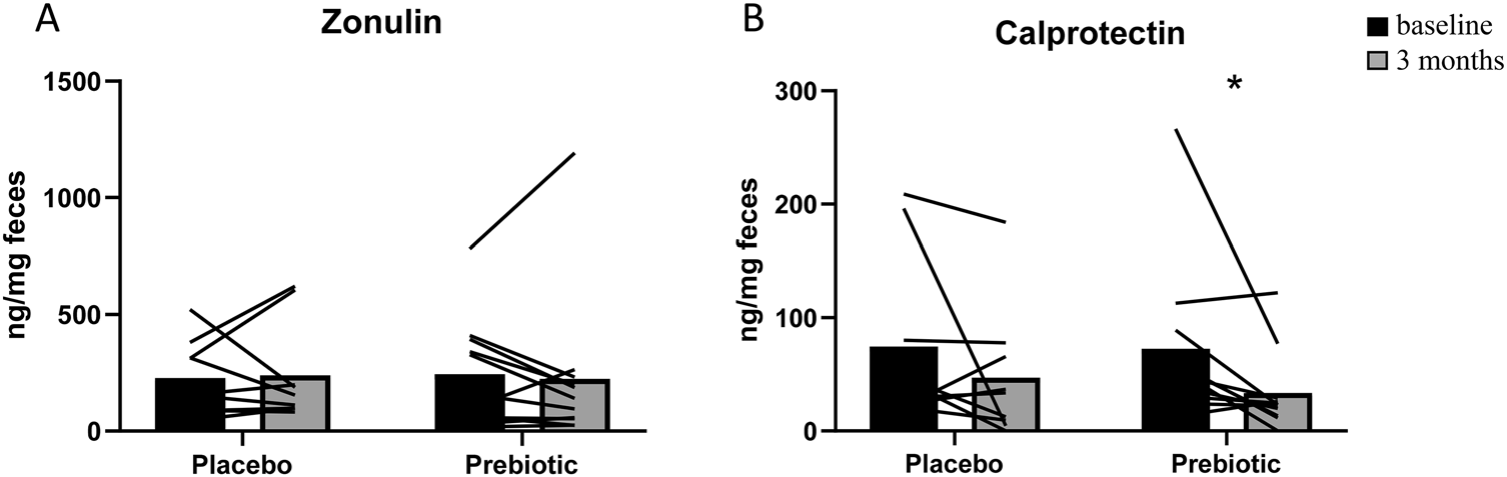
Fecal concentration of zonulin (**a**) and calprotectin (**b**) in obese patients receiving prebiotic or placebo for 3 months. Individual values and means are presented (placebo: *n* = 9; prebiotic: *n* = 11). Baseline data were analyzed by Mann–Whitney test (*p* > 0.05). Matched-pairs Wilcoxon signed-rank tests were performed to compare changes from baseline (within-group variations;*p ≤ 0.05). Between-groups variations were analyzed by Mann–Whitney *U* tests (*p* > 0.05)

We analyzed the impact of the intervention on gut barrier by measuring zonulin in feces, known to regulate tight junctions. High zonulin levels are associated with increased gut permeability [24]. We did not observe any change in fecal zonulin (Fig. 1a). Interestingly, calprotectin, a fecal marker for gut inflammation, decreased of 50% (*p* = 0.019, Wilcoxon test; statistical power = 0.70) after prebiotic intervention (Fig. 1b).

### Fecal short chain fatty acids

**Fig. 2:**
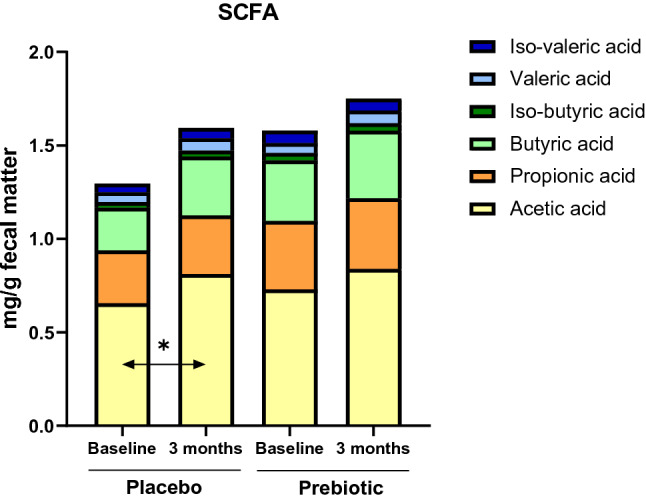
Fecal SCFA profile (% identified SCFA) in obese patients receiving prebiotic or placebo for 3 months. Values are means (placebo: *n* = 10; prebiotic: *n* = 12). Baseline data were analyzed by Mann–Whitney test (*p* > 0.05). Matched-pairs Wilcoxon signed-rank tests were performed to compare changes from baseline (within-group variations;*p ≤ 0.05). Between-groups variations were analyzed by Mann–Whitney *U* tests (*p* > 0.05)

Both interventions increased the total amount of SCFA in fecal samples (but not significantly, *p* > 0.05). Acetate being the major SCFA, significantly increased in the placebo group (statistical power = 0.14) (Fig. 2). Fecal propionic, (iso) butyric and (iso)valeric acid remained unchanged after prebiotic or placebo treatments.

### Fecal bile acids

ITF intake induced minor changes in fecal BA concentrations (Table 1). Although tauro-conjugated BA represented a small proportion of total identified BA (< 1%), an increase in the ratio of tauro-conjugated versus free BA occurred in both placebo and prebiotic groups (statistical power = 0.85 for the prebiotic group); this increase being linked to a higher proportion of taurodeoxycholic acid (TDCA) and taurochenodeoxycholic acid (TCDCA) in the prebiotic group. Of note, we observed a different proportion of TCDCA between both groups before intervention (at baseline).

**Table 1:** Bile acid profile (% identified BA) in obese patients receiving prebiotic or placebo for 3 months^1^

	Placebo				Prebiotic			
		Baseline	3 months	Change		Baseline	3 months	Change
Precursors of CA								
THCA		1.11 ± 0.25	1.01 ± 0.25	−0.09 ± 0.26		0.98 ± 0.24	0.79 ± 0.16	−0.19 ± 0.15
DHCA		0.45 ± 0.16	0.50 ± 0.09	0.05 ± 0.13		0.93 ± 0.37	0.67 ± 0.21	−0.27 ± 0.43
Primary BA								
CA		6.43 ± 2.99	7.99 ± 2.22	1.55 ± 2.05		4.10 ± 1.88	11.45 ± 3.33	7.35 ± 3.85
CDCA		5.52 ± 2.46	8.78 ± 2.56	3.26 ± 3.38		3.69 ± 1.89	8.75 ± 2.17	5.06 ± 3.14
Secondary BA								
UDCA		8.06 ± 4.27	9.84 ± 4.16	1.78 ± 2.13		7.90 ± 2.25	11.40 ± 3.55	3.50 ± 3.75
DCA		69.15 ± 8.22	62.29 ± 6.60	−6.86 ± 5.59		74.11 ± 5.04	59.00 ± 7.20	−15.11 ± 9.04
TDCA		7.55 ± 1.59	7.03 ± 1.04	−0.52 ± 1.46		7.36 ± 0.74	6.25 ± 1.24	−1.11 ± 0.97
Tauro-conjugated BA								
TCA		0.65 ± 0.28	1.26 ± 0.79	0.61 ± 0.54		0.25 ± 0.11	0.49 ± 0.14	0.24 ± 0.15
TCDCA		0.70 ± 0.27	0.70 ± 0.15	−0.00 ± 0.15		0.20 ± 0.08§	0.65 ± 0.18*	0.45 ± 0.17
TDCA		0.39 ± 0.10	0.60 ± 0.11	0.21 ± 0.08		0.48 ± 0.10	0.56 ± 0.11*	0.08 ± 0.15
Total tauro-BA/free BA		0.022 ± 0.007	0.032 ± 0.010*	0.010 ± 0.004		0.011 ± 0.002	0.022 ± 0.003*	0.010 ± 0.00

^1^Values are means ± SEM (placebo: *n*= 8, prebiotic: *n*=9).Baseline data were analyzed by Mann–Whitney test (^§^*p* **<** 0.05 Prebiotic versus Placebo). Matched-pairs Wilcoxon signed-rank tests were performed to compare changes from baseline (within-group variations; *p **<** 0.05). Between-groups variations were analysed by Mann–Whitney *U* tests (*p* > 0.05)

### Fecal long-chain fatty acids

The basal proportion of linolenic acid (C18:3) was different between the groups before intervention (Table 2). Its level increased significantly after ITF treatment whereas it slightly decreased in the placebo group leading to a statistically significant between-variation. More importantly, ITF supplementation significantly increased the levels of rumenic acid (*cis*-9, *trans*-11–18:2) (statistical power = 0.60). Of note, we observed a lower proportion of this conjugated linoleic acid (CLA) in the prebiotic group at baseline.

**Table 2:** Long-chain fatty acid profile (% identified LCFA) in obese patients receiving prebiotic or placebo for 3 months^1^

	Placebo			Prebiotic		
	Baseline	3 months	Change	Baseline	3 months	Change
16:0	26.41 ± 3.34	22.91 ± 2.19	−3.51 ± 2.69	32.88 ± 4.48	26.05 ± 5.41	−6.82 ± 5.91
cis-9-16:1	0.45 ± 0.08	2.19 ± 1.34	1.74 ± 1.30	0.48 ± 0.14	0.46 ± 0.14	−0.02 ± 0.06
18:0	24.86 ± 2.85	18.69 ± 4.20	−6.17 ± 4.45	25.76 ± 3.49	18.57 ± 3.31	−7.18 ± 4.58
trans-9-18:1	0.65 ± 0.23	0.45 ± 0.18	−0.20 ± 0.20	0.86 ± 0.57	0.33 ± 0.11	−0.53 ± 0.53
trans-11-18:1	6.51 ± 1.33	4.29 ± 1.43	−2.22 ± 1.96	3.93 ± 1.04	3.30 ± 0.62	−0.63 ± 0.98
cis-9-18:1	17.14 ± 4.27	29.15 ± 5.54	12.00 ± 7.22	17.52 ± 3.05	15.98 ± 3.17	−1.55 ± 4.29
cis-11-18:1	1.39 ± 0.24	2.39 ± 0.55	1.00 ± 0.63	1.10 ± 0.16	4.97 ± 3.76	3.86 ± 3.65
18:2 (n-6)	15.66 ± 2.87	15.07 ± 3.43	−0.58 ± 3.58	14.69 ± 5.47	23.50 ± 5.04	8.80 ± 7.21
cis-9, trans-11-18:2 (CLA)	0.79 ± 0.36	0.27 ± 0.09	−0.53 ± 0.34	0.08 ± 0.02§	0.37 ± 0.15*	0.28 ± 0.15^#^
trans-10, cis-12-18:2 (CLA)	0.10 ± 0.02	0.05 ± 0.01	−0.05 ± 0.02	0.08 ± 0.02	0.07 ± 0.01	0.00 ± 0.03
18:3 (n-3)	4.56 ± 2.88	3.17 ± 2.36	−1.38 ± 0.69	0.77 ± 0.25§	4.96 ± 1.50*	4.20 ± 1.52^#^
C20:0	0.56 ± 0.07	0.51 ± 0.08	−0.04 ± 0.09	0.64 ± 0.06	0.57 ± 0.10	−0.07 ± 0.11
C20:4 (n-6)	0.13 ± 0.06	0.05 ± 0.01	−0.08 ± 0.06	0.08 ± 0.02	0.07 ± 0.03	0.00 ± 0.04
C22:0	0.43 ± 0.12	0.29 ± 0.04	−0.14 ± 0.11	0.58 ± 0.09	0.48 ± 0.06	−0.10 ± 0.10

^1^Values are means ± SEM (placebo: *n* = 8; prebiotic: *n* = 9). Baseline data were analyzed by Mann-Whitney test (§*p* < 0.05 Prebiotic *versus* Placebo). Matched-pairs Wilcoxon signed-rank tests were performed to compare changes from baseline (within-group variations; *p < 0.05). Between-groups variations were analysed by Mann–Whitney U tests (*p* > 0.05)

### Gut microbiota composition

The alpha-diversity indices related to bacterial richness (Observed species), evenness (Pielou) or both (Shannon), were not significantly affected by the intervention (Fig. S2a). The beta-diversity characterizing overall gut microbiota composition was modified by prebiotic treatment as shown by the PcoA of the Weighted UniFrac distance considering the fraction of branch length in a phylogenic tree (Fig. S2b). In fact, important changes in gut microbiota composition were observed after the prebiotic intervention (Table 3, Fig. S2c). We observed a significant increase in Actinobacteria phylum at the expense of Firmicutes after ITF intake versus placebo. At the family level, it corresponded mostly to changes in abundance of *Bifidobacteriaceae* and *Lachnospiraceae*, respectively to their phyla. Relative abundance of *Lactobacillaceae* also increased after ITF supplementation but to a lesser extent. At the genus level (Table 3), prebiotics largely increased *Bifidobacterium* (statistical power = 0.98)*,* with the change still being significant after adjusting it for multiple testing (*p* = 0.0005, *q* < 0.05). Other weaker changes (at the *p* values) are detailed in Table 3. Among them, placebo intervention increased the relative abundance of *Enterorhabdus*, *Eubacterium* and *Dialister* and decreased *Senegalimassilia*. Prebiotic intervention significantly increased *Anaerostipes* and *Catenibacterium* whereas it decreased *Actinomyces*, *Erysipelotrichaceae* (UCG003) and also 3 unclassified bacteria from family XIII, *Lachnospiraceae* and *Enterobacteriaceae,* respectively. Those genera were not affected by the placebo.

**Table 3:** Bacterial taxa significantly regulated after 3 months of dietary intervention

	Placebo			Prebiotic		
Phylum	Baseline	3 months	Change	Baseline	3 months	Change
Actinobacteria	12.56 ± 1.37	11.15 ± 1.29	−1.41 ± 0.87	12.02 ± 1.32	19.54 ± 1.65 *^q^	7.52 ± 1.53 ^#q^
Firmicutes	69.34 ± 2.66	69.51 ± 2.167	0.17 ± 2.07	70.61 ± 2.21	64.71 ± 2.28 *^q^	−5.90 ± 1.48 ^#^
Family						
Actinomycetaceae	0.36 ± 0.09	0.35 ± 0.06	−0.01 ± 0.11	0.69 ± 0.31	0.35 ± 0.17 *	−0.33 ± 0.16
Bifidobacteriaceae	3.56 ± 1.13	2.81 ± 0.83	−0.75 ± 0.83	4.39 ± 1.06	12.15 ± 1.60 *^q^	7.76 ± 1.33 ^#q^
Lactobacillaceae	0.13 ± 0.09	0.06 ± 0.04	−0.06 ± 0.09	0.13 ± 0.07	1.09 ± 0.47 *	0.96 ± 0.48 ^#^
Family.XIII	0.56 ± 0.18	0.57 ± 0.23	0.01 ± 0.17	0.38 ± 0.12	0.20 ± 0.06 *	−0.18 ± 0.08
Lachnospiraceae	39.01 ± 2.80	37.48 ± 2.59	−1.53 ± 1.47	38.19 ± 2.52	33.32 ± 2.51 *^q^	−4.87 ± 1.24
Ruminococcaceae	19.23 ± 1.66	21.12 ± 1.70 *	1.90 ± 0.70	18.38 ± 1.61	17.11 ± 2.29	−1.27 ± 1.86 ^#^
Enterobacteriaceae	1.75 ± 0.97	1.65 ± 1.18	−0.09 ± 0.33	1.18 ± 0.61	0.52 ± 0.37 *	−0.67 ± 0.29
Erysipelotrichaceae	3.00 ± 0.46	2.53 ± 0.48	−0.47 ± 0.28	2.42 ± 0.40	2.87 ± 0.56	0.47 ± 0.34 ^#^
Genus						
* Actinomyces*	0.35 ± 0.09	0.34 ± 0.06	−0.01 ± 0.11	0.69 ± 0.31	0.35 ± 0.17 *	−0.33 ± 0.16
*Anaerostipes*	1.45 ± 0.42	1.40 ± 0.28	−0.05 ± 0.29	1.38 ± 0.39	3.04 ± 0.71 *	1.65 ± 0.53 ^#^
* Bifidobacterium*	3.56 ± 1.13	2.81 ± 0.83	−0.75 ± 0.83	4.38 ± 1.06	12.15 ± 1.60 *^q^	7.77 ± 1.33 ^#q^
*Catenibacterium*	0.70 ± 0.31	0.60 ± 0.26	−0.10 ± 0.08	0.56 ± 0.26	1.13 ± 0.41 *	0.57 ± 0.20 ^#^
*Dialister*	0.36 ± 0.16	0.23 ± 0.11 *	−0.13 ± 0.05	0.13 ± 0.07	0.3 ± 0.12	0.17 ± 0.11 ^#^
* Enterorhabdus*	0.39 ± 0.139	0.54 ± 0.16 *	0.15 ± 0.06	0.57 ± 0.24	0.71 ± 0.28	0.13 ± 0.19
* Erysipelotrichaceae.UCG.003*	0.67 ± 0.23	0.61 ± 0.18	−0.06 ± 0.12	0.56 ± 0.19	0.37 ± 0.15 *	−0.19 ± 0.08
* Escherichia.Shigella*	0.59 ± 0.26	0.49 ± 0.24	−0.11 ± 0.19	0.67 ± 0.34	0.31 ± 0.22 *	−0.36 ± 0.15
* Eubacterium*	3.98 ± 0.48	5.28 ± 0.36 *	1.30 ± 0.50	4.94 ± 0.98	6.01 ± 1.39	1.07 ± 0.69
* Megamonas*	0.00 ± 0.00	0.00 ± 0.00	0.00 ± 0.00	0.57 ± 0.35	0.99 ± 0.57	0.42 ± 0.25 ^#^
* Senegalimassilia*	0.37 ± 0.09	0.18 ± 0.05 *	−0.19 ± 0.07	0.21 ± 0.12	0.14 ± 0.06	−0.08 ± 0.07
Uncl. Enterobacteriaceae	1.04 ± 0.65	1.03 ± 0.82	−0.01 ± 0.27	0.48 ± 0.27	0.21 ± 0.15 *	−0.27 ± 0.13
Uncl. Family XIII	0.22 ± 0.05	0.23 ± 0.07	0.02 ± 0.06	0.24 ± 0.04	0.09 ± 0.03 *	−0.16 ± 0.04
Uncl. Lachnospiraceae	5.48 ± 0.99	4.06 ± 0.67	−1.42 ± 0.74	5.72 ± 0.78	2.60 ± 0.42 *	−3.12 ± 0.75

Data are expressed as mean percentage of relative abundance and presented as mean ± SEM. Baseline data were analyzed by Mann–Whitney *U* tests (*p* > 0.05). Within-groups variations were analyzed by Wilcoxon matched-pairs test (* *p* < 0.05, FDR correction; ^q^*q*<0.05); between-groups variations were analyzed by Mann–Whitney *U* tests (^#^*p* < 0.05, FDR correction; ^q^*q*<0.05). Uncl, unclassified

### Bacterial genera associated with fecal metabolites

**Fig. 3:**
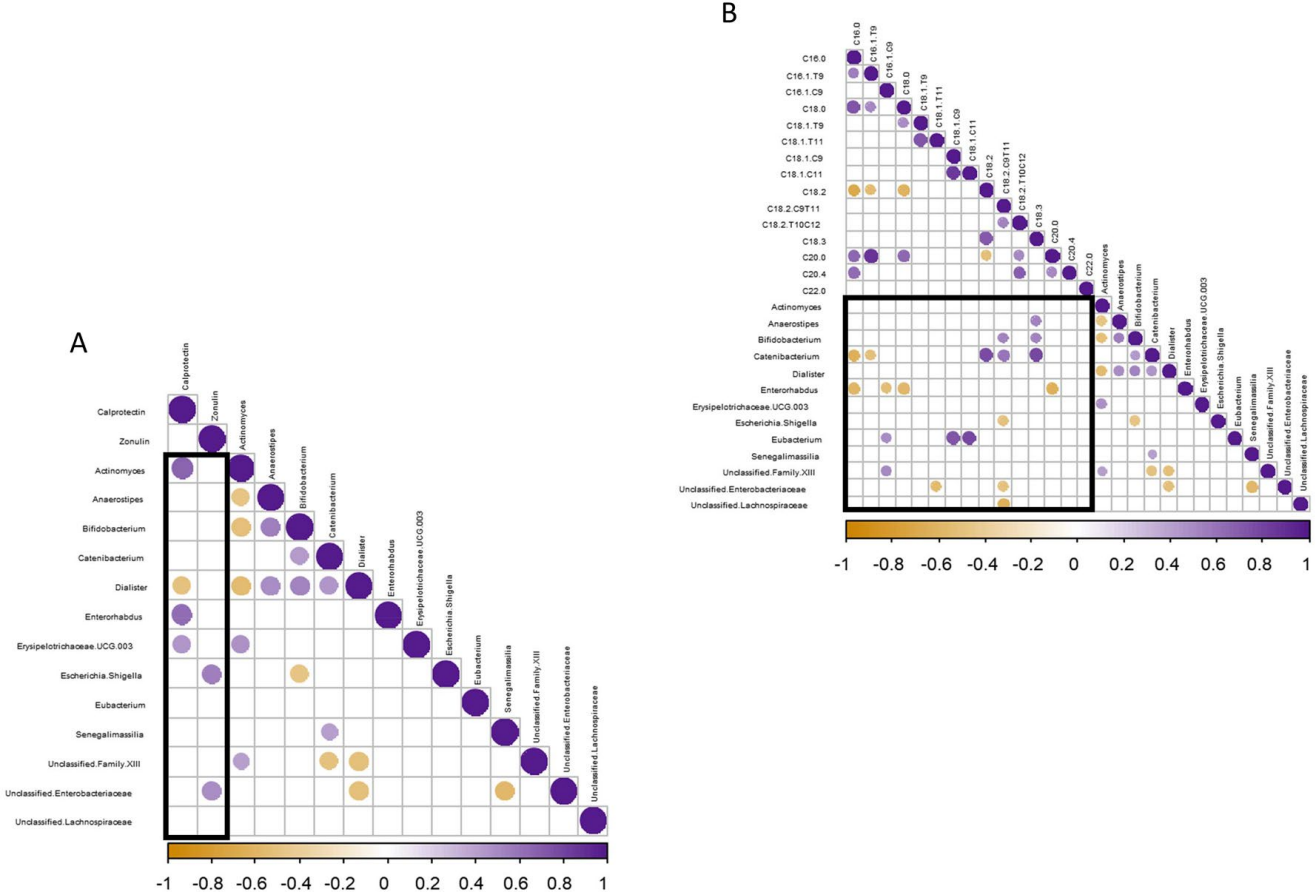
Heatmap of Spearman’s correlations between significant shift in bacteria due to the intervention and the significant shift in fecal concentrations of biomarkers of gut barrier/inflammation (**a**) and of the relative proportions of LCFA (**b**). Orange circles indicate significant negative correlations whereas purple circles represent significant positive correlations (*p* < 0.05)

Changes in fecal calprotectin were negatively correlated with changes in *Dialister* (Fig. 3a). More interestingly, decreased fecal calprotectin was correlated with decreased *Actinomyces* and *Erysipelotrichaceae* (UCG003) (*r* = *0.68 and r* = *0.45*, respectively). We observed that *Erysipelotrichaceae* (UCG003) was the sole bacteria negatively correlated with changes in fecal acetate and positively correlated with changes in THCA, a precursor of cholic acid synthesis (Fig. S3). Importantly, we found that increased rumenic acid (*cis*-9, *trans*-11–18:2) correlated with decreased genera belonging to *Enterobacteriaceae* and higher abundance of *Catenibacterium* and *Bifidobacterium* (*r* =  *− 0.49, p* = *0.04; r* = *0.60*, *p* = 0.01; and *r* = *0.52, p* = *0.03* respectively, Fig. 3b).

## Discussion

We have previously shown that improvement of anthropometric and cardiometabolic risk parameters of obese patients after prebiotic intervention may be related to specific changes in gut bacteria (i.e., a decrease in *Desulfovibrio* and *Clostridium* sensu stricto) in the whole FOOD4GUT cohort [22]. Here, although their caloric intake was significantly reduced in both groups (by 14% and 12% for placebo and prebiotic groups, respectively), anthropometric and cardiometabolic risk parameters were not significantly affected by the intervention, probably due to the limited number of patients in this subcohort. We cannot exclude that dietary intake reporting has been underestimated, an effect frequently observed in obese patients and that can explain discrepancies between energy intake ad body weight. Such discrepancy has already been observed in similar study [32]. Interestingly, we observed that fructan intake was much higher in the patients receiving cookbook based on ingredients rich in fructan (prebiotic group).

Intestinal microbiota alterations in obese subjects have already been associated with local and systemic inflammation, suggesting that obesity-related microbiota have a proinflammatory effect [33]. Despite the limited number of patients per group, our data revealed that prebiotic intervention decreased fecal calprotectin of obese patients with a substantial statistical power. Although ITF supplementation does not influence serum markers of inflammation [22], our results suggest that it decreases local gut inflammation, an effect which could be interesting in patients presenting co-morbidities like diverticulosis associated with overweight or obesity [34]. It is interesting that the statistical drop in one species of *Erysipelotrichaceae* in the prebiotic group was associated with lowered calprotectin*. Erysipelotrichacea*e, belongs to the Firmicutes phylum and was correlated with gastrointestinal tract inflammation in patients with colorectal cancer or inflammatory bowel disease [35]. Higher levels of *Erysipelotrichaceae* have been found in obese individuals, their levels being dependent on the type of dietary fat [35].

SCFA are major products of bacterial fermentation of ITF. There are controversies concerning their link with obesity [1, 36–38]. In our previous study performed in obese women, SCFA significantly decreased after the prebiotic ITF treatment and acetate (among other SCFA) positively correlated with BMI, fasting insulinemia and HOMA index, suggesting that SCFA might be involved in body weight increase and insulin resistance [39]. In view of data comparing lower body weight evolution and fat mass development in germ-free mice with conventionalized animals, the energy harvest hypothesis has been developed, leading to the idea that SCFA may promote adiposity by different mechanisms [2]. The lack of effect on fecal SCFA in obese patients after prebiotic intake in this exploratory study may be linked to the small number of fecal samples available for SCFA analysis leading to a too low statistical power. Despite this limitation, we observed that acetate levels increased significantly in the placebo group. It is important to consider that majority of SCFA (up to 95%) are rapidly absorbed by the colonocytes resulting in decreasing concentrations from the proximal to distal colon. Therefore, only a minor fraction of SCFA (about 5%) is excreted in faeces [40]. Thus, we cannot conclude that the higher SCFA level strictly reflects SCFA production by the gut microbiota.

We did not detect any differences in primary BA profile after prebiotic intake. The BA profile in the colon is mainly unconjugated along with secondary BA, notably due to the action of bile salt hydrolases (BSH) that hydrolyze conjugated BA to free BA and glycine or taurine [41]. BSH may be found in *Clostridium, Enterococcus, Bifidobacterium, Lactobacillus* and *Bacteroides* [42]. Although it concerns BA in very low proportion in stool samples (< 1% of total BA) as previously described [43], we demonstrated that the proportion of tauro-conjugated BA *versus* free BA increased in both groups, in favor of TDCA and TCDCA after prebiotic intervention. No correlation was found between bacteria modulated by the intervention and those BA. Overall, the data obtained in this cohort do not support these metabolites as biomarkers reflecting the interaction between prebiotic DF and the gut microbiota in obese patients.

PUFA may be also reduced by bacteria, leading to trans- and conjugated-fatty acids. We have previously shown the ability of the gut microbiota to produce PUFA-derived metabolites from dietary PUFA germ-free *versus* conventionalized mice [10]. In humans, we demonstrated that gut microbial metabolites of PUFA correlate with specific fecal bacteria and serum markers of metabolic syndrome in obese women [44]. CLA and conjugated linolenic acids (CLnA) derive from the biohydrogenation of linoleic acid (C18:2) and linolenic acid (C18:3) of bacteria that express linole(n)ic acid isomerase. Of these, rumenic acid (*cis*-9, *trans*-11–18:2) is most naturally abundant, representing approximately 85% of all naturally occurring CLA isomers. It is associated with positive health benefits, especially demonstrated in human studies with obesity metrics as an endpoint [12, 45, 46]. We observed an increase of linolenic acid (C18:3) in fecal material after prebiotic intake that may be related to dietary intake of n-3 PUFA. More importantly, we observed an increase of rumenic acid in parallel to an increase of bifidobacteria after 3 months of prebiotic supplementation. In addition, the higher proportion of fecal rumenic acid correlated with bifidogenic effect after ITF intervention*.* It was already shown in vitro that bifidobacteria incubated with linoleic acid in deuterium oxide-enriched medium formed labelled rumenic acid [46]. Of particular interest, the improvement of gut barrier (regulation of tight junction proteins) and gut inflammation (lower inflammatory cytokines in colonic tissues) by CLA (mixture of isomers composed 50% of *cis*-9, *trans*-11 CLA) have been demonstrated (in a dose-dependent manner) in a mouse model of colitis [45]. Penedo et al*.* [47] showed that the intake of a *cis*-9, *trans*-11 CLA-enriched butter by normal-weight subjects induces beneficial changes in immune modulators associated with sub-clinical inflammation in overweight individuals. Although the contribution of dietary supplementation *versus* the gut microbial production of rumenic acid remains to be established, our results together with other studies suggested that the increase of rumenic acid could be considered as a biomarker of ITF interaction with gut microbiota through its bifidogenic effect.

In conclusion, the drop in fecal calprotectin observed after prebiotic intake emphasizes the potential interest of prebiotic intake to combat gut inflammatory disorders occurring with obesity, an effect that could be related to changes in the abundance of bacteria like *Erysipelotrichaceae*. If the increase in *Bifidobacterium* appears as a reproducible signature of inulin intake in the whole cohort of obese individuals, other bacteria or bacterial co-metabolites (like rumenic acid) could be implicated in ITF interactions with the gut microbiota and have relevance for health.

## Supplementary Information

Below is the link to the electronic supplementary material.

Supplementary file1 (DOCX 289 KB)

## References

[1] Vallianou N, Stratigou T, Christodoulatos GS, Dalamaga M (2019). Understanding the role of the gut microbiome and microbial metabolites in obesity and obesity-associated metabolic disorders: current evidence and perspectives. Curr Obes Rep.

[2] Koh A, De Vadder F, Kovatcheva-Datchary P, Backhed F (2016). From dietary fiber to host physiology: short-chain fatty acids as key bacterial metabolites. Cell.

[3] Dalile B, Van Oudenhove L, Vervliet B, Verbeke K (2019). The role of short-chain fatty acids in microbiota-gut-brain communication. Nat Rev Gastroenterol Hepatol.

[4] Delzenne NM, Cani PD (2011). Interaction between obesity and the gut microbiota: relevance in nutrition. AnnuRevNutr.

[5] Delzenne NM, Neyrinck AM, Cani PD (2011). Modulation of the gut microbiota by nutrients with prebiotic properties : consequences for host health in the context of obesity and metabolic syndrome. Microb Cell Fact.

[6] Delzenne NM, Cani PD, Everard A, Neyrinck AM, Bindels LB (2015). Gut microorganisms as promising targets for the management of type 2 diabetes. Diabetol Clin Ex Diabetes Meta.

[7] Delzenne NM, Rodriguez J, Olivares M, Neyrinck AM (2020). Microbiome response to diet: focus on obesity and related diseases. Rev Endocr Metab Disord.

[8] Chavez-Talavera O, Tailleux A, Lefebvre P, Staels B (2017). Bile acid control of metabolism and inflammation in obesity, Type 2 diabetes, dyslipidemia, and nonalcoholic fatty liver disease. Gastroenterology.

[9] Vincent RP, Omar S, Ghozlan S, Taylor DR, Cross G, Sherwood RA, Fandriks L, Olbers T, Werling M, Alaghband-Zadeh J, le Roux CW (2013). Higher circulating bile acid concentrations in obese patients with type 2 diabetes. Ann Clin Biochem.

[10] Druart C, Bindels LB, Schmaltz R, Neyrinck AM, Cani PD, Walter J, Ramer-Tait AE, Delzenne NM (2015). Ability of the gut microbiota to produce PUFA-derived bacterial metabolites: proof of concept in germ-free versus conventionalized mice. Mol Nutr Food Res.

[11] Moya-Camarena SY, Vanden Heuvel JP, Blanchard SG, Leesnitzer LA, Belury MA (1999). Conjugated linoleic acid is a potent naturally occurring ligand and activator of PPARalpha. J Lipid Res.

[12] den Hartigh LJ (2019). Conjugated linoleic acid effects on cancer, obesity, and atherosclerosis: a review of pre-clinical and human trials with current perspectives. Nutrients.

[13] Toomey S, McMonagle J, Roche HM (2006). Conjugated linoleic acid: a functional nutrient in the different pathophysiological components of the metabolic syndrome?. Curr Opin Clin Nutr Metab Care.

[14] Gibson GR, Hutkins R, Sanders ME, Prescott SL, Reimer RA, Salminen SJ, Scott K, Stanton C, Swanson KS, Cani PD, Verbeke K, Reid G (2017). Expert consensus document: the international scientific association for probiotics and prebiotics (ISAPP) consensus statement on the definition and scope of prebiotics. Nat Rev Gastroenterol Hepatol.

[15] Gibson GR, Roberfroid MB (1995). Dietary modulation of the human colonic microbiota: introducing the concept of prebiotics. J Nutr.

[16] Delzenne NM, Olivares M, Neyrinck AM, Beaumont M, Kjolbaek L, Larsen TM, Benitez-Paez A, Romani-Perez M, Garcia-Campayo V, Bosscher D, Sanz Y, van der Kamp JW (2020). Nutritional interest of dietary fiber and prebiotics in obesity: lessons from the MyNewGut consortium. Clin Nutr.

[17] Delzenne NM, Neyrinck AM, Cani PD (2011). Modulation of the gut microbiota by nutrients with prebiotic properties: consequences for host health in the context of obesity and metabolic syndrome. Microb Cell Fact.

[18] Delzenne NM, Neyrinck AM, Backhed F, Cani PD (2011). Targeting gut microbiota in obesity: effects of prebiotics and probiotics. Nat Rev Endocrinol.

[19] Green M, Arora K, Prakash S (2020). Microbial medicine: prebiotic and probiotic functional foods to target obesity and metabolic syndrome. Int J Mol Sci.

[20] Vallianou N, Stratigou T, Christodoulatos GS, Tsigalou C, Dalamaga M (2020). Probiotics, prebiotics, synbiotics, postbiotics, and obesity: current evidence, controversies, and perspectives. Curr Obes Rep.

[21] Cerdo T, Garcia-Santos JA (2019). The role of probiotics and prebiotics in the prevention and treatment of obesity. Nutrients.

[22] Hiel S, Gianfrancesco MA, Rodriguez J, Portheault D, Leyrolle Q, Bindels LB, da Silveira G, Cauduro C, Mulders M, Zamariola G, Azzi AS, Kalala G, Pachikian BD, Amadieu C, Neyrinck AM, Loumaye A, Cani PD, Lanthier N, Trefois P, Klein O, Luminet O, Bindelle J, Paquot N, Cnop M, Thissen JP, Delzenne NM (2020). Link between gut microbiota and health outcomes in inulin-treated obese patients: Lessons from the Food4Gut multicenter randomized placebo-controlled trial. Clin Nutr.

[23] Neyrinck AM, Rodriguez J, Vinoy S, Maquet V, Walter J, Bischoff SC, Laville M, Delzenne NM (2020). The FiberTAG project: tagging dietary fibre intake by measuring biomarkers related to the gut microbiota and their interest for health. Nutr Bull.

[24] Schwiertz A, Spiegel J, Dillmann U, Grundmann D, Burmann J, Fassbender K, Schafer KH, Unger MM (2018). Fecal markers of intestinal inflammation and intestinal permeability are elevated in Parkinson's disease. Parkinsonism Relat Disord.

[25] Fasano A (2020). All disease begins in the (leaky) gut: role of zonulin-mediated gut permeability in the pathogenesis of some chronic inflammatory diseases. F1000Res.

[26] Roseth AG, Schmidt PN, Fagerhol MK (1999). Correlation between faecal excretion of indium-111-labelled granulocytes and calprotectin, a granulocyte marker protein, in patients with inflammatory bowel disease. Scand J Gastroenterol.

[27] Kalala G, Kambashi B, Everaert N, Beckers Y, Richel A, Pachikian B, Neyrinck AM, Delzenne NM, Bindelle J (2018). Characterization of fructans and dietary fibre profiles in raw and steamed vegetables. Int J Food Sci Nutr.

[28] Neyrinck AM, Nazare JA, Rodriguez J, Jottard R, Dib S, Sothier M, Berghe LVD, Alligier M, Alexiou H, Maquet V, Vinoy S, Bischoff SC, Walter J, Laville M, Delzenne NM (2020). Development of a repertoire and a food frequency questionnaire for estimating dietary fiber intake considering prebiotics: input from the FiberTAG project. Nutrients.

[29] Rodriguez J, Neyrinck AM, Zhang Z, Seethaler B, Nazare JA, Robles Sanchez C, Roumain M, Muccioli GG, Bindels LB, Cani PD, Maquet V, Laville M, Bischoff SC, Walter J, Delzenne NM (2020). Metabolite profiling reveals the interaction of chitin-glucan with the gut microbiota. Gut Microbes.

[30] Bolyen E, Rideout JR, Dillon MR, Bokulich NA, Abnet CC, Al-Ghalith GA, Alexander H, Alm EJ, Arumugam M, Asnicar F, Bai Y, Bisanz JE, Bittinger K, Brejnrod A, Brislawn CJ, Brown CT, Callahan BJ, Caraballo-Rodriguez AM, Chase J, Cope EK, Da Silva R, Diener C, Dorrestein PC, Douglas GM, Durall DM, Duvallet C, Edwardson CF, Ernst M, Estaki M, Fouquier J, Gauglitz JM, Gibbons SM, Gibson DL, Gonzalez A, Gorlick K, Guo J, Hillmann B, Holmes S, Holste H, Huttenhower C, Huttley GA, Janssen S, Jarmusch AK, Jiang L, Kaehler BD, Kang KB, Keefe CR, Keim P, Kelley ST, Knights D, Koester I, Kosciolek T, Kreps J, Langille MGI, Lee J, Ley R, Liu YX, Loftfield E, Lozupone C, Maher M, Marotz C, Martin BD, McDonald D, McIver LJ, Melnik AV, Metcalf JL, Morgan SC, Morton JT, Naimey AT, Navas-Molina JA, Nothias LF, Orchanian SB, Pearson T, Peoples SL, Petras D, Preuss ML, Pruesse E, Rasmussen LB, Rivers A, Robeson MS, Rosenthal P, Segata N, Shaffer M, Shiffer A, Sinha R, Song SJ, Spear JR, Swafford AD, Thompson LR, Torres PJ, Trinh P, Tripathi A, Turnbaugh PJ, Ul-Hasan S, van der Hooft JJJ, Vargas F, Vazquez-Baeza Y, Vogtmann E, von Hippel M, Walters W, Wan Y, Wang M, Warren J, Weber KC, Williamson CHD, Willis AD, Xu ZZ, Zaneveld JR, Zhang Y, Zhu Q, Knight R, Caporaso JG (2019). Reproducible, interactive, scalable and extensible microbiome data science using QIIME 2. Nat Biotechnol.

[31] Benjamini YHY (1995). Controlling the false discovery rate: a practical and powerful approach to multiple testing. J Roy Stat Soc.

[32] Parnell JA, Reimer RA (2009). Weight loss during oligofructose supplementation is associated with decreased ghrelin and increased peptide YY in overweight and obese adults. Am J Clin Nutr.

[33] Verdam FJ, Fuentes S, de Jonge C, Zoetendal EG, Erbil R, Greve JW, Buurman WA, de Vos WM, Rensen SS (2013). Human intestinal microbiota composition is associated with local and systemic inflammation in obesity. Obesity (Silver Spring).

[34] Murray KA, Hoad CL, Garratt J, Kaviani M, Marciani L, Smith JK, Siegmund B, Gowland PA, Humes DJ, Spiller RC (2019). A pilot study of visceral fat and its association with adipokines, stool calprotectin and symptoms in patients with diverticulosis. PLoS ONE.

[35] Kaakoush NO (2015). Insights into the role of erysipelotrichaceae in the human host. Front Cell Infect Microbiol.

[36] Schwiertz A, Taras D, Schafer K, Beijer S, Bos NA, Donus C, Hardt PD (2010). Microbiota and SCFA in lean and overweight healthy subjects. Obesity (Silver Spring).

[37] Fava F, Gitau R, Griffin BA, Gibson GR, Tuohy KM, Lovegrove JA (2013). The type and quantity of dietary fat and carbohydrate alter faecal microbiome and short-chain fatty acid excretion in a metabolic syndrome 'at-risk' population. Int J Obes (Lond).

[38] Teixeira TF, Grzeskowiak L, Franceschini SC, Bressan J, Ferreira CL, Peluzio MC (2013). Higher level of faecal SCFA in women correlates with metabolic syndrome risk factors. Br J Nutr.

[39] Salazar N, Dewulf EM, Neyrinck AM, Bindels LB, Cani PD, Mahillon J, de Vos WM, Thissen JP, Gueimonde M, de Los Reyes-Gavilan CG, Delzenne NM (2015). Inulin-type fructans modulate intestinal Bifidobacterium species populations and decrease fecal short-chain fatty acids in obese women. Clin Nutr.

[40] Verbeke KA, Boobis AR, Chiodini A, Edwards CA, Franck A, Kleerebezem M, Nauta A, Raes J, van Tol EA, Tuohy KM (2015). Towards microbial fermentation metabolites as markers for health benefits of prebiotics. Nutr Res Rev.

[41] Ridlon JM, Kang DJ, Hylemon PB (2006). Bile salt biotransformations by human intestinal bacteria. J Lipid Res.

[42] Zeng H, Umar S, Rust B, Lazarova D, Bordonaro M (2019). Secondary bile acids and short chain fatty acids in the colon: a focus on colonic microbiome, cell proliferation, inflammation, and cancer. Int J Mol Sci.

[43] Humbert L, Maubert MA, Wolf C, Duboc H, Mahe M, Farabos D, Seksik P, Mallet JM, Trugnan G, Masliah J, Rainteau D (2012). Bile acid profiling in human biological samples: comparison of extraction procedures and application to normal and cholestatic patients. J Chromatogr B Analyt Technol Biomed Life Sci.

[44] Druart C, Dewulf EM, Cani PD, Neyrinck AM, Thissen JP, Delzenne NM (2014). Gut microbial metabolites of polyunsaturated fatty acids correlate with specific fecal bacteria and serum markers of metabolic syndrome in obese women. Lipids.

[45] Chen Y, Yang B, Ross RP, Jin Y, Stanton C, Zhao J, Zhang H, Chen W (2019). Orally administered CLA Ameliorates DSS-induced colitis in mice via intestinal barrier improvement, oxidative stress reduction, and inflammatory cytokine and gut microbiota modulation. J Agric Food Chem.

[46] McIntosh FM, Shingfield KJ, Devillard E, Russell WR, Wallace RJ (2009). Mechanism of conjugated linoleic acid and vaccenic acid formation in human faecal suspensions and pure cultures of intestinal bacteria. Microbiology.

[47] Penedo LA, Nunes JC, Gama MA, Leite PE, Quirico-Santos TF, Torres AG (2013). Intake of butter naturally enriched with cis9, trans11 conjugated linoleic acid reduces systemic inflammatory mediators in healthy young adults. J Nutr Biochem.

