# Supplementary material for: Prebiotic dietary fibre intervention improves fecal markers related to inflammation in obese patients: results from the Food4Gut randomized placebo-controlled trial
Source: Eur J Nutr. 2021 Feb 5;60(6):3159–70. doi: 10.1007/s00394-021-02484-5 (PMC8354918; doi:10.1007/s00394-021-02484-5)
Supplement: Supplementary file 1 — Supplementary file1 (DOCX 289 KB) [file 394_2021_2484_MOESM1_ESM.docx]

**ELECTRONIC SUPPLEMENTARY MATERIAL**

**Title:** Prebiotic dietary fibre intervention improves fecal markers related to inflammation in obese patients: results from the Food4Gut randomized placebo-controlled trial

**Authors:** Audrey M. Neyrinck^1#^, Julie Rodriguez^1#^, Zhengxiao Zhang^2^, Benjamin Seethaler^3^, Cándido Robles Sánchez^1^, Martin Roumain^4^, Sophie Hiel^1^, Laure B. Bindels^1^, Patrice D. Cani^1,5^, Nicolas Paquot^6^, Miriam Cnop^7,8^, Julie-Anne Nazare^9^, Martine Laville^9^, Giulio G. Muccioli^4^, Stephan C. Bischoff^3^, Jens Walter^2,10^, Jean-Paul Thissen^11^ and Nathalie M. Delzenne^1*^

**Author affiliations**

^1^Metabolism and Nutrition Research Group, Louvain Drug Research Institute, UCLouvain, Université catholique de Louvain, Belgium

^2^Department of Medicine, University of Alberta, Edmonton, Canada

^3^Institute of Nutritional Medicine, University of Hohenheim, Germany

^4^Bioanalysis and Pharmacology of Bioactive Lipids Research Group, Louvain Drug Research Institute, UCLouvain, Université catholique de Louvain, Belgium.

^5^WELBIO- Walloon Excellence in Life Sciences and BIOtechnology, UCLouvain, Université catholique de Louvain, Brussels, Belgium.

^6^Laboratory of Diabetology, Nutrition and Metabolic disease, Liège, Université de Liège, Belgium;

^7^ULB Center for Diabetes Research, Université Libre de Bruxelles ; ^8^Division of Endocrinology, Erasmus Hospital, Université Libre de Bruxelles, Brussels, Belgium;

^9^Rhône-Alpes Research Center for Human Nutrition, Université-Lyon, CarMeN Laboratory, Hospices Civils de Lyon, France

^10^APC Microbiome Ireland, Department of Medicine, and School of Microbiology, University College Cork, Cork, Ireland

^11^Pole of Endocrinology, Diabetes and Nutrition, Institut de Recherche Expérimentale et clinique, UCLouvain, Université catholique de Louvain, Brussels, Belgium

^#^These authors contributed equally to this work.

**Corresponding author:** Prof. Nathalie M. Delzenne, Metabolism and Nutrition Research Group, Louvain Drug Research Institute, UCLouvain, Université catholique de Louvain, avenue E. Mounier box B1.73.11, B-1200 Brussels, Belgium; E-mail address: nathalie.delzenne@uclouvain.be; Phone: +32 2 764 73 69

**Table S1**. Anthropometric and cardiometabolic risk factors in obese patients receiving prebiotic or placebo for 3 months^1^

|  | **Placebo** | | | | | | **Prebiotic** | | | | | |
| --- | --- | --- | --- | --- | --- | --- | --- | --- | --- | --- | --- | --- |
|  | Baseline | | | 3 months | | | Baseline | | | 3 months | | |
|  |  |  |  |  |  |  |  |  |  |  |  |  |
| Body weight, kg | 105 | ± | 4 | 104 | ± | 3 | 104 | ± | 5 | 102 | ± | 6 |
| BMI, kg/m² | 35 | ± | 2 | 35 | ± | 2 | 36 | ± | 1 | 36 | ± | 1 |
| Fat mass, kg | 34 | ± | 4 | 34 | ± | 3 | 39 | ± | 3 | 38 | ± | 3 |
| Waist, cm | 116 | ± | 3 | 114 | ± | 2 | 113 | ± | 3 | 112 | ± | 3 |
| SBP, mm Hg | 138 | ± | 4 | 130 | ± | 5 | 134 | ± | 3^§^ | 135 | ± | 4 |
| DBP, mm Hg | 85 | ± | 3 | 83 | ± | 2 | 85 | ± | 2 | 86 | ± | 3 |
| Total chol, mg/dl | 169 | ± | 18 | 168 | ± | 17 | 194 | ± | 17 | 199 | ± | 22 |
| LDL-chol, mg/dl | 89 | ± | 15 | 88 | ± | 14 | 114 | ± | 14 | 118 | ± | 18 |
| HDL-chol, mg/dl | 45 | ± | 2 | 48 | ± | 4 | 47 | ± | 2 | 47 | ± | 3 |
| TG, mg/dl | 177 | ± | 28 | 157 | ± | 24 | 163 | ± | 26 | 168 | ± | 32 |
| Glycemia, mg/dl | 119 | ± | 8 | 116 | ± | 11 | 100 | ± | 6 | 101 | ± | 5 |
| Insulin, mU/L | 18 | ± | 2 | 15 | ± | 2 | 18 | ± | 3 | 16 | ± | 3 |
| HbA1c, % | 6.3 | ± | 0.3 | 6.1 | ± | 0.2 | 5.9 | ± | 0.2 | 5.9 | ± | 0.2 |
| HOMA-IR | 5.3 | ± | 0.8 | 4.3 | ± | 0.6 | 4.7 | ± | 1.0 | 4.4 | ± | 0.9 |
| CRP, mg/l | 3641 | ± | 1785 | 1792 | ± | 510 | 3166 | ± | 917 | 4750 | ± | 1542 |

^1^Values are means ± SEM (placebo: n = 12; prebiotic: n = 12). Baseline data were analyzed by Mann-Whitney test (^§^p < 0.05 Prebiotic versus Placebo). Matched-pairs Wilcoxon signed-rank tests were performed to compare changes from baseline (within-group variations; p **>** 0.05). Between-groups variations were analysed by Mann–Whitney U-tests (p > 0.05). BMI, body mass index; Chol, cholesterol; CRP, C-reactive protein; DBP, diastolic blood pressure; HDL, High-density lipoprotein; HOMA-IR, homeostasis model assessment of insulin resistance; LDL, Low-density lipoprotein; SBP, systolic blood pressure; TG, Triglyceride.

**Fig S1.** Daily energy intakes in obese patients receiving prebiotic or placebo for 3 months. Values are means ± SEM (placebo: n = 11; prebiotic: n = 12). Baseline data were analyzed by Mann-Whitney test (p > 0.05 Prebiotic *versus* Placebo). Matched-pairs Wilcoxon signed-rank tests were performed to compare changes from baseline (within-group variations; *p **<** 0.05). Between-groups variations were analysed by Mann–Whitney U-tests (p > 0.05).

**Table S2.** Daily nutrient intakes in obese patients receiving prebiotic or placebo for 3 months^1^

|  | **Placebo** | | | | | | | | | **Prebiotic** | | | | | | | | |
| --- | --- | --- | --- | --- | --- | --- | --- | --- | --- | --- | --- | --- | --- | --- | --- | --- | --- | --- |
|  | Baseline | | | 3 months | | | **Change** | | | Baseline | | | 3 months | | | **Change** | | |
| *Energy (kcal)* | 2171 | ± | 133 | 1862 | ± | 104* | **-308** | **±** | **76** | 1863 | ± | 102 | 1636 | ± | 118* | **-227** | **±** | **83** |
| *Protein (g)* | 91 | ± | 6 | 82 | ± | 5* | **-9** | **±** | **3** | 85 | ± | 4 | 73 | ± | 4* | **-12** | **±** | **4** |
| *Carbohydrates (g)* | 215 | ± | 13 | 196 | ± | 19 | **-19** | **±** | **12** | 202 | ± | 14 | 180 | ± | 19 | **-22** | **±** | **14** |
| Sugars (g) | 82 | ± | 8 | 62 | ± | 8* | **-19** | **±** | **6** | 65 | ± | 7 | 73 | ± | 9 | **8** | **±** | **8^#^** |
| Starch (g) | 129 | ± | 10 | 131 | ± | 15 | **3** | **±** | **12** | 134 | ± | 9 | 106 | ± | 12* | **-28** | **±** | **10^#^** |
| *Fat (g)* | 88.1 | ± | 9.4 | 69.8 | ± | 5.4* | **-18.3** | **±** | **5.3** | 71.3 | ± | 5.5 | 61.8 | ± | 5.1 | **-9.5** | **±** | **4.9** |
| SFA (g) | 32.3 | ± | 5.0 | 26.4 | ± | 3.2* | **-5.8** | **±** | **2.5** | 27.7 | ± | 3.1 | 23.0 | ± | 2.5* | **-4.6** | **±** | **2.2** |
| MUFA (g) | 29.9 | ± | 3.6 | 25.3 | ± | 2.9 | **-4.6** | **±** | **1.9** | 18.4 | ± | 2.9^§^ | 16.7 | ± | 2.9 | **-1.8** | **±** | **3.4** |
| PUFA (g) | 11.7 | ± | 2.9 | 7.5 | ± | 0.9 | **-4.2** | **±** | **2.5** | 5.9 | ± | 0.7^§^ | 7.1 | ± | 1.7 | **1.2** | **±** | **1.5** |
| PUFA n-3 (g) | 1.5 | ± | 0.3 | 1.0 | ± | 0.3* | **-0.5** | **±** | **0.2** | 0.9 | ± | 0.1 | 1.2 | ± | 0.3 | **0.3** | **±** | **0.3** |
| PUFA n-6 (g) | 8.5 | ± | 2.6 | 5.0 | ± | 0.7* | **-3.5** | **±** | **2.1** | 4.1 | ± | 0.6^§^ | 5.3 | ± | 1.3 | **1.1** | **±** | **1.2** |
| *trans-*FA (g) | 1.3 | ± | 0.4 | 0.7 | ± | 0.1* | **-0.6** | **±** | **0.3** | 0.8 | ± | 0.1 | 0.7 | ± | 0.1 | **-0.1** | **±** | **0.1** |
| Cholesterol (mg) | 223 | ± | 32 | 187 | ± | 21 | **-36** | **±** | **20** | 176 | ± | 19 | 135 | ± | 17* | **-40** | **±** | **16** |
| *Fibre (g)* | 25 | ± | 2 | 24 | ± | 2 | **-1** | **±** | **2** | 23 | ± | 3 | 25 | ± | 3 | **2** | **±** | **3** |
| Fructan (g) | 2.2 | ± | 0.4 | 2.7 | ± | 0.4 | **0.5** | ± | **0.5** | 2.9 | ± | 0.5 | 9.3 | ± | 2.2* | **6.3** | ± | **2.2°** |
|  |  |  |  |  |  |  |  |  |  |  |  |  |  |  |  |  |  |  |

^1^Values are means ± SEM (placebo: n = 11; prebiotic: n = 12). Baseline data were analyzed by Mann-Whitney test (^§^p < 0.05 Prebiotic *versus* Placebo). Matched-pairs Wilcoxon signed-rank tests were performed to compare changes from baseline (within-group variations; *p **<** 0.05). Between-groups variations were analysed by Mann–Whitney U-tests (^#^p **<** 0.05; °p = 0.059).


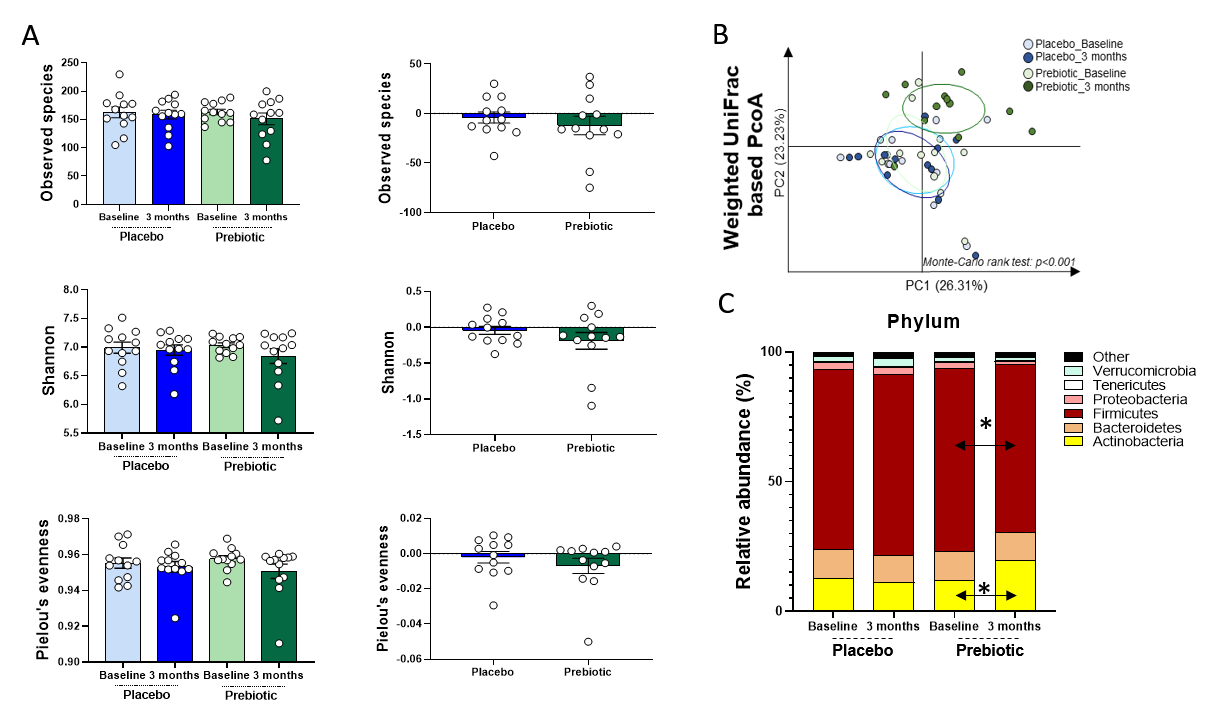


**Fig S2.** Overall composition of the gut microbiota in obese patients receiving prebiotic or placebo for 3 months. (**a**) Measures of alpha-diversity: Observed species, Pielou’s evenness measure and Shannon. Data are expressed as mean ± SEM. (**b**) Principal coordinates analysis (PCoA) of the beta-diversity index (Weighted UniFrac). p-values refer to Monte Carlo rank test performed on R software. (**c**) Barplots of percentage in the mean relative abundance of phyla accounting for more than 1%. Matched-pairs Wilcoxon signed-rank tests were performed to compare changes from baseline (within-group variations; *p < 0.05). Between-groups variations were analysed by Mann–Whitney U-tests (p > 0.05).


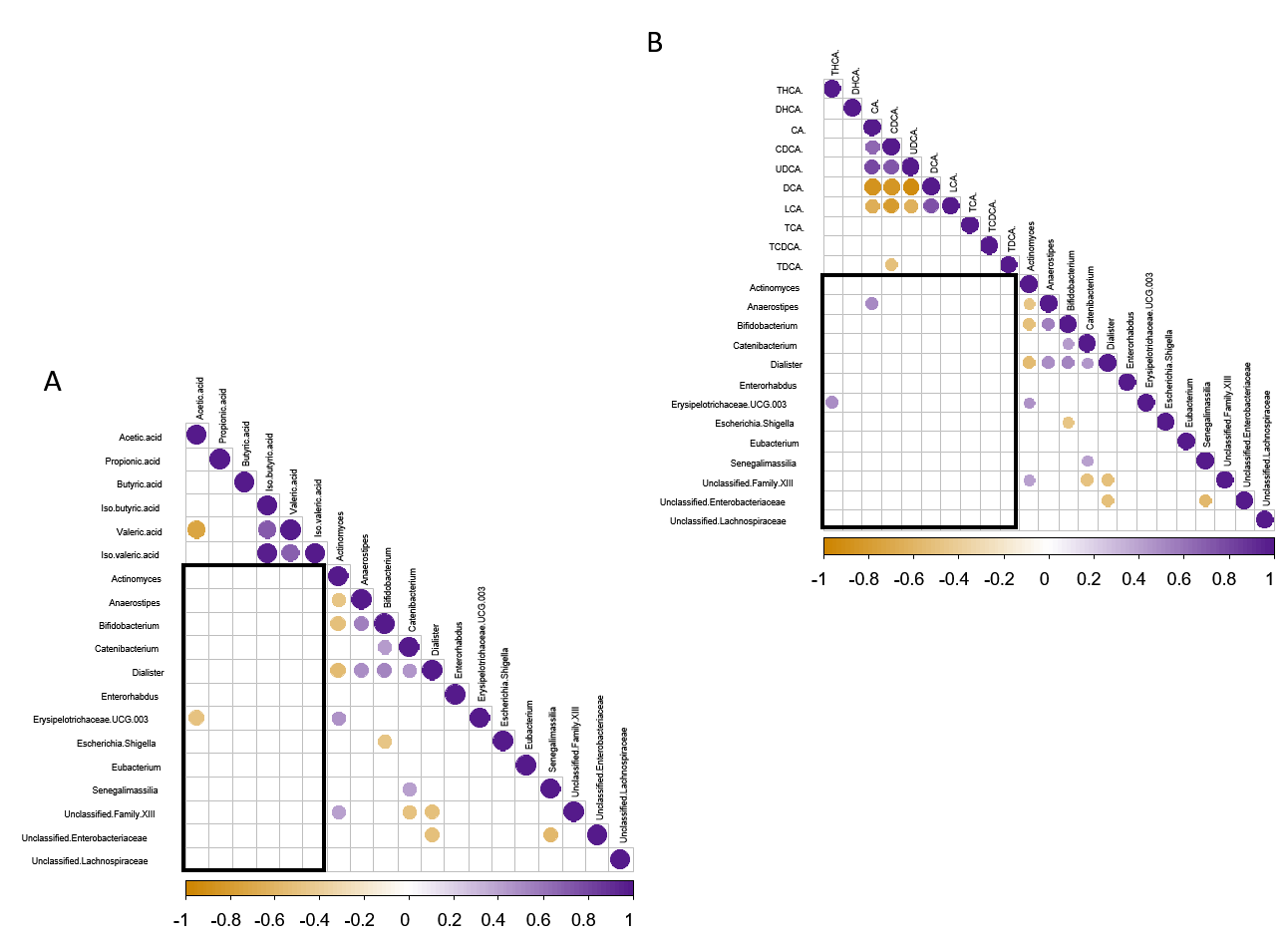


**Fig S3.** Heatmap of Spearman’s correlations between significant shift in bacteria due to intervention and the significant changes in the proportion of fecal SCFA (**a**) and of fecal BA (**b**). Orange circles indicate significant negative correlations whereas purple circles represent significant positive correlations (p < 0.05).
